# Improving Carotenoid Extraction from Tomato Waste by Pulsed Electric Fields

**DOI:** 10.3389/fnut.2014.00012

**Published:** 2014-08-12

**Authors:** Elisa Luengo, Ignacio Álvarez, Javier Raso

**Affiliations:** ^1^Tecnología de los Alimentos, Facultad de Veterinaria, Universidad de Zaragoza, Zaragoza, Spain

**Keywords:** carotenoids, extraction, tomato, PEF, by-product

## Abstract

In this investigation, the influence of the application of pulsed electric fields (PEFs) of different intensities (3–7 kV/cm and 0–300 μs) on the carotenoid extraction from tomato peel and pulp in a mixture of hexane:acetone:ethanol was studied with the aim of increasing extraction yield or reducing the percentage of the less green solvents in the extraction medium. According to the cellular disintegration index, the optimum treatment time for the permeabilization of tomato peel and pulp at different electric field strengths was 90 μs. The PEF permeabilization of tomato pulp did not significantly increase the carotenoid extraction. However, a PEF treatment at 5 kV/cm improved the carotenoid extraction from tomato peel by 39% as compared with the control in a mixture of hexane:ethanol:acetone (50:25:25). Further increments of electric field from 5 to 7 kV/cm did not increase significantly the extraction of carotenoids. The presence of acetone in the solvent mixture did not positively affect the carotenoid extraction when the tomato peels were PEF-treated. Response surface methodology was used to determine the potential of PEF for reducing the percentage of hexane in a hexane:ethanol mixture. The application of a PEF treatment allowed reducing the hexane percentage from 45 to 30% without affecting the carotenoid extraction yield. The antioxidant capacity of the extracts obtained from tomato peel was correlated with the carotenoid concentration and it was not affected by the PEF treatment.

## Introduction

The tomato is one of the most widely cultivated vegetable crops. Millions of tomato tons are processed every year to produce products such as ketchup and sauce, resulting in large amounts of by-products, such as peel, pulp, and seeds that represent a 10–40% of total processed tomatoes. Around 70% of wet pomace consists of the skin and pulp that are lycopene-rich components of waste originate from tomato paste manufacturing (1). Therefore, the tomato and its processed food products are considered to be one of the best sources of lycopene. Lycopene is the principal carotenoid in tomato that causes the fruit’s characteristically red color. This compound can represent approximately 80–90% of the total carotenoids in the tomato (2). Lycopene can be used as a coloring and antioxidant agent in the food industry, and it is also used as a nutraceutical because of, its high antioxidant activity, thus reducing the risk of atherosclerosis and coronary heart disease. Moreover, epidemiological studies have connected the intake of lycopene to a lower risk of the incidence of certain types of cancers (2). Therefore, extracting lycopene from tomato waste is a good alternative for the valorization of this by-product.

The extraction of carotenoids from vegetable sources is usually carried out by using organic solvents (e.g., hexane, acetone, chloroform, ethanol, etc.) because they are soluble in fat. A mixture of hexane with acetone and ethanol is often employed (2, 3). Also, supercritical fluids using non-organic solvents are suitable for the extraction of compounds that can easily become degraded by light, oxygen, and high temperatures like lycopene, but the solubility of these substances is still relatively low compared to their solubility in organic solvents. High pressures must be applied to obtain reasonable extraction from dried vegetable material, making it a costlier process (4). Consequently, from an industrial point of view, solvent extraction is the first option because of its simplicity and low costs. However, the process is very time consuming and requires large amounts of solvents according to the mass of the final products.

In the extraction of natural products, the use of solvents defines a major part of the environmental performance, cost, safety, and health issues of the process. Studies performed by Capello et al. (5) have shown that hexane and acetone have higher combined environment, health, and safety (EHS) risk scores than ethanol.

Improving and optimization of the existing process and innovation in process and procedures are solutions that have been suggested for the design of green and sustainable methods to extract of natural products (6). Hence, there is an increasing demand for extraction techniques that improve extraction yield with a shorter extraction time and reduced organic solvent consumption (7). The ability of several methods, such as enzymes or ultrasounds, to assist in the extraction of lycopene from the tomato has been evaluated by different authors (8–10). These techniques require the sample to be ground and on occasions dried before the extraction. Such operations may cause a loss of the lycopene content and an increase in the processing cost.

Pulsed electric fields (PEFs) assisted extraction has been shown to be a promising technology for improving the extraction of valuable compounds from soft fresh vegetable materials (11). The process is based on the application of external electric fields that induce the electroporation of cytoplasmic membranes, thus enhancing the diffusion of solutes located inside the cells. This permeabilization of cell membranes can be achieved at moderate electric fields (<10 kV/cm) and low specific energies (<10 kJ/kg).

An enhancement in the extraction of hydrophilic compounds such as sugar from the sugar beet, betaine from the red bet, and anthocyanins from grapes, red cabbage, or purple fleshed potatoes (12–14) through the application of a PEF treatment has been reported. However, the efficacy of PEF in improving the extraction of fat-soluble compounds has scarcely been investigated (15).

The key advantages of PEF-assisted extraction are that it is a non-thermal treatment that does not affect the quality of the extracted products, and the fact that it is possible to apply the treatments in a continuous flow, both at pilot plant and on an industrial scale, to fresh material (16).

In this investigation, it was studied the influence of the application of PEFs of different intensities (3–7 kV/cm and 0–300 μs) on the carotenoid extraction from tomato peel and pulp in a mixture of hexane:acetone:ethanol with the aim of increasing extraction yield or reducing the percentage of solvents with a higher EHS. Response surface methodology (RSM) was used to determine the potential of PEF for reducing the percentage of hexane in a hexane:ethanol mixture without affecting the carotenoid extraction yield (CEY) and the antioxidant capacity of the extracts.

## Materials and Methods

### Plant material

Red tomatoes (commercial variety: *tomate canario*) were purchased from a local supermarket and stored at 4°C until required. Color measurements were performed on the surface of the tomatoes, at least four times, with a Minolta Chroma Meter CR-200 (Minolta Camera Co., Ltd., Osaka, Japan) tristimulus color analyzer. The readings were obtained in the CIE L*a*b* color space and the coordinates L*, a*, and b* were obtained by using the D65 standard observer and a visual angle of 10°. Tomatoes with similar coordinates were selected in order to use fruit that exhibited a homogeneous carotenoid concentration (17). Tomatoes were hand peeled. The resulting peel was cut in pieces with a diameter of 24 mm; the pulp was cut into pieces with a diameter of 15 mm.

### Chemicals

Analytical-grade hexane and ethanol, analytical grade, were purchased from VWR International (Fontenay-sous-Bois, France). All solvents for HPLC analysis (acetonitrile, hexane, and methanol) were of a HPLC gradient grade and were obtained from Fisher Scientific (Fair Lawn, NJ, USA). All-trans-lycopene was purchased from Sigma Chemical Co. (Sigma-Aldrich Company, St. Louis, MO, USA).

### PEF treatment

The PEF equipment that was used in this investigation was supplied by ScandiNova (Modulator PG, ScandiNova, Uppsala, Sweden). The apparatus generates square waveform pulses of a width of 3 μs with a frequency of up to 300 Hz. The maximum output voltage and current were 30 kV and 200 A, respectively. The equipment consists of a direct current power supply, which converts the 3-phase line voltage to a regulated DC voltage. It charges up 6 IGBT switching modules (high-power solid-state switches) to a primary voltage around 1000 V. An external trigger pulse gates all the modules and controls its discharge to a primary pulsed signal of around 1000 V. Finally, a pulse transformer converts this primary 1000 V pulse to the desired high-voltage pulse.

The treatment chambers consisted of a cylindrical methacrylate tube closed with two polished stainless steel cylinders. Two different size chambers were used in this study due to the differences in the conductivity of peel and pulp. In order to treat the peel, a chamber with an electrode diameter of 24 mm and a gap of 5 mm was used. For the pulp treatment, the electrode diameter was 15 mm and the gap was 10 mm.

The actual voltage and current intensity that were applied were measured with a high-voltage probe (Tektronix, P6015A, Wilsonville, OR, USA) and a current probe (Stangenes Industries, Inc., Palo Alto, CA, USA), respectively that were connected to an oscilloscope (Tektronix, TDS 220, Wilsonville, OR, USA).

The PEF treatments that ranged from 5 to 100 pulses of 3 μs (45–300 μs), set at electric field strength ranging from 3 to 7 kV/cm were used. The specific energy of these treatments ranged from 0.54 to 13.50 kJ/kg. A pulse frequency of 1 Hz was used.

### Cell disintegration index

The cell disintegration index (*Z_p_*) was used to identify the PEF treatment conditions for the pre-treatment of the tomato peel or pulp before the carotenoid extraction. This index characterizes the proportion of permeabilized cells based on the frequency dependence of conductivity of intact and permeabilized plant tissues (18).

The cell disintegration index analysis was carried out by using impedance measurement equipment (DIL, Quakenbrück, Germany). For the experiments, untreated and PEF-treated disks of tomato peel or pulp were placed in the measuring cell of the equipment. *Z_p_* was calculated by using the following equation:

(1) $$Z_{p}=1-\left(\frac{K_{h}}{K_{h}^{\prime }}\right)\cdot \frac{\left(K_{h}^{\prime }-K_{l}^{\prime }\right)}{\left(K_{h}-K_{l}\right)};\;0\leq Z_{p}\leq 1$$

where *K*_1_, $K_{l}^{\prime }$ are the electrical conductivities of untreated and PEF-treated material, respectively, at a low-frequency field (1–5 kHz); *K_h_*, $K_{h}^{\prime }$ are the electrical conductivities of untreated and PEF-treated material, respectively, at a high-frequency field (3–50 MHz). The *Z_p_* varies between 0 for intact tissues and 1 for a tissue with all the cells permeabilized.

### Carotenoid extraction

Carotenoids were extracted by using different organic solvents and solvent mixtures. The polar solvents that were used consisted of acetone and ethanol, while the non-polar solvent that was used was hexane. A mixture of hexane:acetone:ethanol [50:25:25 (v:v:v)] was used in order to establish the intensity of the electric field strength to obtain the highest extraction. The first series of experiments were conducted with single solvents or mixtures of equal volumes (50:50) at a solvent to waste ratio of 20:1 (v:w). The second series of experiments were conducted with three mixtures of hexane and ethanol 25:75, 50:50, and 75:25 (v:v).

Six disks (5.0 ± 0.5 g) of the untreated and PEF-treated tomato peel, or four disks (5.0 ± 0.5 g) of the untreated and PEF-treated tomato pulp were put in a 250 mL Erlenmeyer flask that contained 100 mL of the solvent. The flasks were incubated at 25°C in a water bath and shaken at 120 rpm. Samples of 1 mL of the extraction solvent were removed at different extraction times (10, 20, 40, 60, 90, 120, 150, 180, 210, 240, 270, and 300 min).

### Carotenoid quantification

The extracts that were obtained at different points in time were centrifuged at 5,400 × *g* for 6 min to separate the supernatant. When hexane was one of the components of the mixture of solvents that were used for extraction, the presence of water in the sample permitted to separate the extracting solution into poorly differentiated polar and non-polar layers. Adding 0.1 mL of water to 1 mL of the supernatant caused a complete separation into distinct polar and non-polar layers. The absorbance of the non-polar layer (i.e., the hexane layer) containing lycopene was measured at 472 nm on a spectrophotometer (Jenway 6505 UV/VIS, Jenway, Felsted, UK). Absolute hexane was used as blank. The amount of carotenoids was determined by using the molar extinction coefficient of lycopene in hexane at 472 nm (E1% 1 cm 3450) (19) and expressed as milligram of carotenoids/100 g of fresh weight (FW) tomato peel or pulp.

### HPLC analysis of carotenoids

Before the HPLC analysis, the extracts were concentrated on a miVac concentrator (GeneVac Ltd., UK) for 15 min at 30°C by vacuum evaporation of 10 mL of the hexane and re-dissolved in 2 mL of hexane.

HPLC/DAD analyses were performed on a Varian ProStar high performance liquid chromatograph (Varian Inc., Walnut Creek, CA, USA) that was equipped with a ProStar 240 ternary pump, a ProStar 410 AutoSampler, and a ProStar 335 photodiode array detector. The system was controlled with a Star chromatography workstation v.6.41 (Varian). A reversed-phase column Microsorb-MV 100-5 C18 (25 cm × 0.46 cm; 5 μm particle size) with a precolumn (5 cm × 0.46 cm; 5 μm particle size) of the same material was used. The temperature of the column and the precolumn was maintained at 30°C.

A linear gradient that consisted of acetonitrile (A), hexane (B), and methanol (C) was used as follows: 70% A, 7% B, and 23% C to 70% A, 4% B, and 26% C within 10 min. The flow rate through the column was 1.5 mL/min, the sample injection was 10 μL, and the absorbance detection wavelength was 472 nm. Prior to injection, all of the samples were filtered through a 0.2 μm sterile syringe filter of cellulose acetate (VWR, West Chester, PA, USA).

Lycopene was identified by comparing their retention time and visible absorption spectra with this of its standard. Lutein and β-carotene were identified according to their retention time and characteristic absorption spectra found in bibliography (20–22).

### Antioxidant capacity

The antioxidant activity of the extracts was measured by using the modified ABTS [2,2′ azinobis (3-ethylbenzothiazoline-6-sulfonic acid) diammonium salt] radical decolorization assay (23). The ABTS radical was generated by adding 2 g of MnO_2_ into 100 mL of 5 mL ABTS solution, stirred for 20 min at room temperature and filtered through a 0.2 PTFE syringe filter. On the day of analysis, the ABTS solution was diluted with ethanol (96%) to an absorbance of 0.700 ± 0.02 at 734 nm. Then 100 μL of the extract was added to 1 mL of the ABTS radical solution and vortexed for 10 s. One minute after the addition of the sample, the decolourization that was caused by reduction of the cations by antioxidants from the sample was measured spectrophotometrically at 734 nm (Jenway 6505 UV/VIS, Jenway, Felsted, UK). Assays were performed in triplicate. Trolox^®^ (6 hydroxy-2,5,7,8-trimethyl-chroman-2-carboxylic acid), a water-soluble vitamin E analog, was used to prepare the standard curve and the antioxidant activity was reported as micrograms of Trolox equivalent antioxidant.

### Experimental design

Response surface methodology was used to determine optimal PEF-assisted extraction of carotenoids from tomato peel with respect to the hexane percentage in a hexane:ethanol solvent mixture and extraction time. Preliminary experiments indicated that an electric field of 5 kV/cm and treatment time of 90 μs were the optimal PEF treatment conditions. Therefore, these PEF treatment conditions were selected to compare the CEY from untreated and PEF-treated tomato peel. A central composite design (CCD) was constructed to investigate the effects of hexane:ethanol solvent (from 25 to 75% of hexane) and extraction time (from 0 to 300 min) on CEY. The obtained data were modeled with the following second-order polynomial equation:

(2) $$Y=\beta_{0}+\sum_{i=1}^{k}\beta_{i}X_{i}+\sum_{i=1}^{k}\beta_{ii}{X_{i}}^{\text{2}}+\sum_{i>1}^{k}\beta_{ij}X_{i}X_{j}$$

where *Y* is the response variable to be modeled, *X_i_* and *X_j_* are independent factors, β_0_ is the intercept, β*_i_* is the linear coefficients, β*_ij_* is the quadratic coefficients, β*_ij_* is the cross-product coefficients, and *k* is the total number of independent factors. A backward regression procedure was used to determine the parameters of the models. This procedure systematically removed the effects that were not significantly associated (*p* > 0.05) with the response until a model with only a significant effect was obtained.

The CCD and the corresponding analysis of the data were carried out by using the software package Design-Expert 6.0.6 (Stat-Ease Inc., Minneapolis, MN, USA).

### Statistical analysis

Experiments were performed in triplicate and the presented results are means ± SD. One-way analysis of variance (ANOVA) using Tukey’s test was performed to evaluate the significance of differences between the means values. The differences were considered significant at *p* < 0.05. GraphPad PRISM (GraphPad Software, San Diego, CA, USA) was used to perform the statistical analysis.

## Results and Discussion

### Characterization of the PEF induced damage in cells of tomato peel

**Figure 1:**
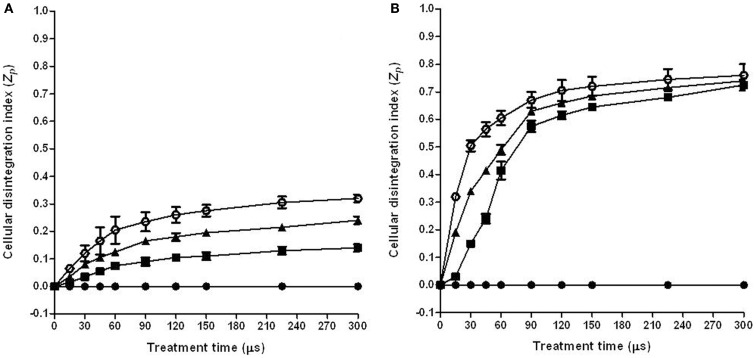
**Influence of electric field strength and treatment time on the cell disintegration index (*Z_p_*) of tomato peel (A) and pulp (B)**. (●) Control, (■) 3 kV/cm, (▲) 5 kV/cm, (○) 7 kV/cm. The error bars represent SEM.

The *Z_p_* was used to select the optimum PEF treatment conditions to permeabilize the cells of the tomato peel and pulp. This index, which is based on the changes of conductivity of intact and PEF permeabilization tissue, has been used by different authors in different vegetable tissues for this purpose (24). Figure 1 shows the influence of the treatment time on the *Z_p_* of tomato peel (Figure 1A) and pulp (Figure 1B) at different electric field strengths.

The general trend on the influence of the electric field strength and treatment time on the *Z_p_* value that was observed in this research supports previously reported data for other vegetable tissues such as potato, apple, onion, or orange (18, 25, 26).

The increase in electric field strength and treatment time resulted in an increment of the *Z_p_* to a highest value of around 0.7 for the pulp and 0.3 for the peel. These results indicate that higher electric field strengths are required to permeabilization tomato peel cells than pulp cells. These noticeable differences to the electroporation effect between both tissues could be related to the cell size. As the transmembrane potential induced by PEF is proportional to the cell radius, lower electric field strength is required to induce the electroporation of pulp cells because they are bigger than peel cells (27).

Although the effect of the electric field strength on *Z_p_* was different for both tissues, in both cases the *Z_p_* value significantly increased with the treatment time up to 90 μs (30 pulses of 3 μs), independent of the electric field strength that was applied. Further increments of the treatment time barely affected to the *Z_p_* value. According to these results, in additional studies that aimed to investigate the influence of electric field strength on the extraction of carotenoids, the treatment time was set at 90 μs.

### Characterization of the PEF treatment in the extraction of lycopene from tomato peel

**Figure 2:**
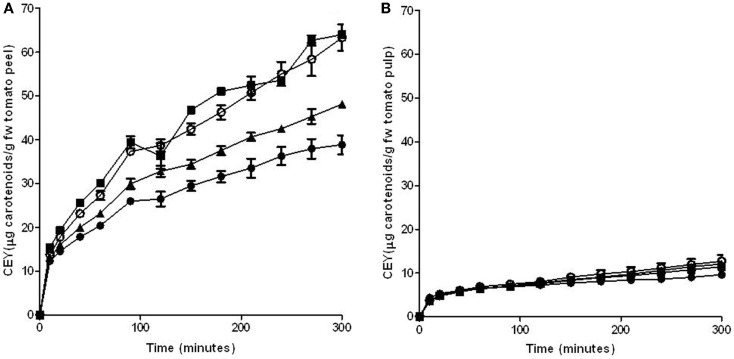
**Influence of electric field strength on the carotenoid extraction yield (CEY) from tomato peel (A) and pulp (B) using a mixture of hexane:acetone:ethanol (50:25:25) as extraction solvent**. (●) Control, (■) 3 kV/cm, (▲) 5 kV/cm, (○) 7 kV/cm. The error bars represent SEM.

A mixture of hexane with acetone and ethanol has been used by different authors to extract carotenoids from tomatoes and tomato products (2, 28, 29). The effect of the application of PEF treatments on improving the extraction of carotenoids from tomato peel and pulp was tested in this solvent mixture. Figure 2 shows the influence of a PEF treatment application at different electric field strengths (3, 5, and 7 kV/cm for 90 μs) on the extraction of carotenoids from peel (Figure 2A) and pulp (Figure 2B) through the time.

The results obtained in this investigation confirm the observations of other authors who have reported that tomato skin contained significantly higher amounts of lycopene than tomato pulp (30). Moreover, while the extraction of lycopene from the peel increased with the intensity of the electric field strength that was applied, the application of PEF treatments of different intensities to the pulp did not significantly increase the lycopene extraction as in comparison to the untreated sample. Therefore, the permeabilization of the cells of the pulp by PEF did not affect either the capacity of penetration of the solvent into the cells or the diffusion of carotenoids through the cytoplasmic membrane, resulting in a similar extraction from both intact and permeabilized cells. For this reason, no further experiments were made with this matrix.

On the contrary, it can be observed that the extraction of carotenoids from tomato peels was significantly improved by the application of the PEF treatment. This increment was augmented by increasing the electric field strength up to 5 kV/cm. However, an increase in the intensity of the electric field strength up to 7 kV/cm did not involve any further rise in the CEY. After 200 min, the extraction of carotenoids from tomato peels improved 13 and 39% in comparison to the control when the peels had been PEF-treated with 3 and 5 kV/cm, respectively. Therefore, the electric field strength of 5 kV/cm was set for further experimentations.

The increase in the carotenoids extraction yield can be explained by the electroporation effect caused by PEF on the cytoplasmic membrane of tomato peel cells. The increment of the permeability of the cytoplasmic membrane that acts as a semipermeable barrier, facilitates the penetration of the solvents into the cells and the release of the carotenoids located inside the cells, and it increases the carotenoid extraction rate. For instance, the application of a PEF treatment at 5 kV/cm reduced the extraction time for obtaining the same amount of carotenoids (690 μg/100g) in the untreated and PEF-treated samples from 200 to 85 min.

### Effect of the PEF treatment in the extraction of lycopene from tomato peel with different solvents

**Figure 3:**
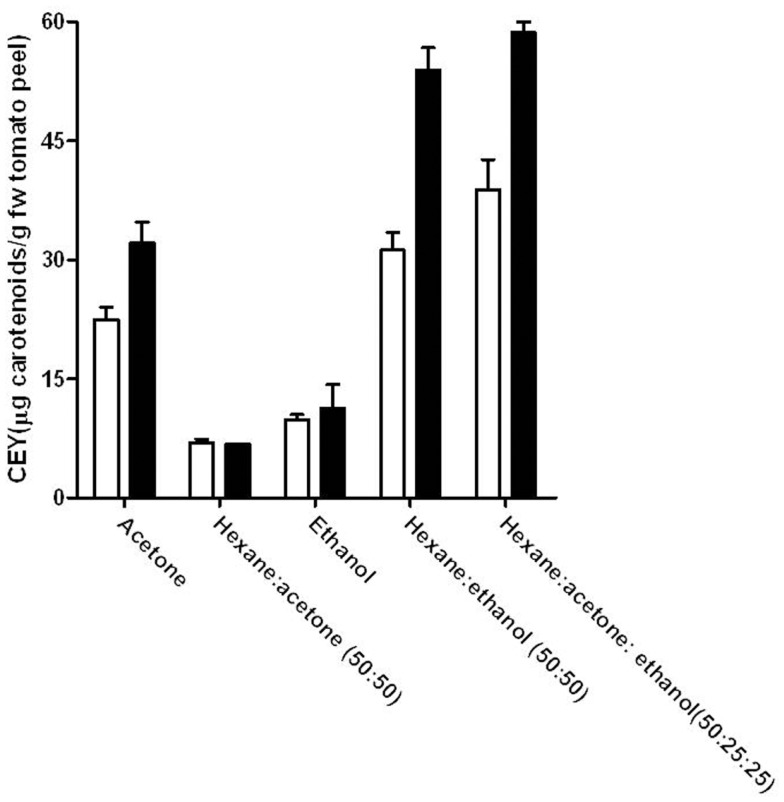
**Influence of the solvent extraction mixture on the carotenoid extraction yield (CEY) from control (white bars) and PEF-treated (5 kV/cm–90 μs) (black bars) tomato peel after 300 min of extraction**. The error bars represent SEM.

Figure 3 compares the CEY for untreated and PEF-treated (5 kV/cm, 90 μs) samples of tomato peel by using different individual, or mixture, of solvents after 300 min of extractions. The combination of hexane with ethanol or with ethanol and acetone improved the total yield in comparison to that obtained by any of the individual solvents. On the contrary, acetone alone presented a higher CEY than its mixture with hexane. The solubility capacity of solvents plays a very important role in the extraction process, even in the samples that were previously treated by PEF. Ethanol is a polar solvent that hardly solubilizes carotenoids, due to their lipophilicity. Nevertheless, the combination of ethanol and a non-polar solvent such as hexane considerably improved the extraction of carotenoids. Therefore, the combination of ethanol with hexane shows a synergistic effect. In addition to the solubility, the capacity of penetration or diffusion of the solvents into the solid matrix also has an important role in the extraction efficiency. Acetone alone is a good solvent and a wetting material that penetrates easier in the solid matrix than the hexane:acetone mixture. In this case, the addition of hexane reduced the acetone capacity to penetrate into the cells, and the yield obtained by the mixture was lower than that of acetone in both untreated and PEF-treated samples. Similar results were obtained by Strati (31), who found that the acetone:hexane mixture was less efficient than acetone in extracting carotenoids from dry tomato waste, whereas Lin (32) found that the hexane:ethanol mixture was also more efficient than the hexane:acetone mixture in extracting carotenoids from tomato juice. The presence of water in the tomato juice and the higher solubility of ethanol than acetone in water could explain this fact. The importance of solvent solubility in water, when extraction is conducted in a fresh matrix, was demonstrated by the lack of extraction of carotenoids in both untreated and PEF-treated samples when hexane was used as solvent (results not shown).

The application of a PEF treatment to the tomato peels before extraction involved an increase of the CEY when the extraction was made with acetone, hexane:ethanol (50:50), or hexane:acetone:ethanol (50:25:25), whereas no significant effect of the PEF treatment was observed when the extraction was made with hexane:acetone (50:50) or ethanol. Therefore, although the permeabilization of the tomato peel cells has been demonstrated (Figure 1), the use of solvents with enough solubility and penetration capacity is necessary to observe an improvement in the CEY in comparison to the control.

The highest carotenoid yield (38 and 58 μg/g of FW tomato peel from untreated and PEF-treated tomato peel, respectively) was obtained when carotenoids were extracted with a mixture of hexane:acetone:ethanol (50:25:25). However, no significant differences (*p* > 0.05) were observed between the extraction with this mixture and the hexane:ethanol mixture when a PEF treatment was applied prior to extraction. As (EHS) risk scores for acetone are higher than those for ethanol in further experiments, acetone was removed from the solvent mixture and the optimization of the hexane and ethanol mixture was carried out.

### Optimization of solvent content in the mixture hexane:ethanol for PEF-assisted extraction of carotenoids

The CEY resulting from the three different proportions of hexane:ethanol (25:75, 50:50, 75:25) at different extraction times (20, 160, 300 min) for the control (untreated) and the PEF-treated tomato peels are shown in Table 1. The experimental values of CEY there were obtained varied from 6.48 to 58.81 μg/g FW tomato peel. These contents are within the range of the values that are reported in the literature by other authors who have investigated the extraction of carotenoids from fresh tomato waste in different solvents and solvent mixtures at distinct temperatures (29, 33, 34).

**Table 1:** **Effect of percentage of hexane and extraction time on the carotenoid extraction yield (CEY) from tomato peel non-treated (control) and PEF-treated**.

Hexane (%)	Time (min)	CEY (μg/g FW tomato peel)	
		Control	PEF-treated
25	20	11.80 ± 0.65	12.29 ± 0.20
	160	24.21 ± 0.89	27.87 ± 2.05^a^
	300	31.61 ± 1.32	39.00 ± 0.92^a^
50	20	18.24 ± 3.53	19.54 ± 3.27
	160	30.01 ± 3.02	40.85 ± 7.23^a^
	300	44.14 ± 4.00	58.81 ± 9.44^a^
75	20	6.78 ± 0.48	6.48 ± 0.53
	160	9.43 ± 0.61	11.80 ± 1.43
	300	11.54 ± 0.15	15.62 ± 0.89^a^

*The results are expressed as mean ± CL (95%)*.

*^a^Significantly differences (*p* < 0.05) between the extraction from control and PEF-treated peels*.

The permeabilization of the tomato peel by PEF prior to the extraction process did not significantly increase the CEY (*p* > 0.05) in the first extraction times. However, for longer extraction times, the CEY improved until it reached approximately 30% in comparison to the control by the application of the PEF treatment.

**Figure 4:**
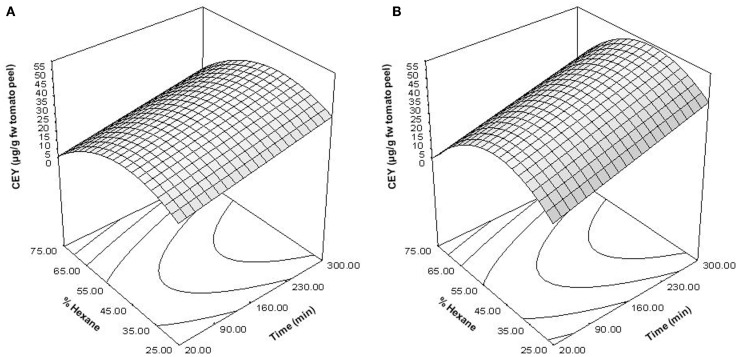
**Response surface plots showing the influence of the percentage of hexane and extraction time on the carotenoid extraction yield (CEY) expressed as microgram of carotenoids per gram of fresh weight (FW) tomato peel**. Control **(A)**; PEF-treated **(B)**.

In order to optimize the hexane percentage and extraction time required for the extraction of carotenoids from tomato peel treated by PEF, a multiple regression analysis was performed according to the experimental data shown in Table 1. The analysis resulted in second-order polynomial equations for both untreated and PEF-treated samples after removing the non-significant terms (*p* > 0.05). Figure 4 shows the response surface graphs obtained with these polynomial equations. Table 2 indicates the coefficients and *F*-values for significant variables and their interactions for equations describing the relationship between the CEY, the percentage of hexane in the solvent and the extraction time for both untreated and PEF-treated samples. A summary of the ANOVA for the selected quadratic models is also shown in Table 2.

**Table 2:** ***F*-value and *p*-value of significant variables and their interaction of the polynomial equation describing the influence of the percentage of hexane and extraction time on the carotenoid extraction yield (CEY) from tomato peel non-treated (control) and PEF-treated**.

	Control			PEF		
	
	Coefficient	*F*-value	*p*-Value	Coefficient	*F*-value	*p*-Value
Intercept	−25.71			−44.71		
H	+2.156	16.43	0.0098	3.15	9.75	0.0168
*t*	+0.0606	10.07	0.0247	+0.089	26.59	0.0013
H^2^	−0.0242	17.39	0.0087	−0.034	36.22	0.0005
Model		14.63	0.0066		29.93	0.0005
*R*^2^		0.89			0.91	
*R*^2^-adj		0.83			0.87	
RMSE		0.00			0.	
Lack of fit		26.37			49.05	

The determination coefficient for each model was higher than 0.90, which means that <10% of the total response variation remained unexplained by the models. The adjusted *R*^2^ (*R*^2^-adj) values, which correct the *R*^2^ according to the sample size and the number of terms in the model (35), were similar to the corresponding *R*^2^, indicating that there is good agreement between the experimental and predicted values. This statement was confirmed according to the root mean square error (RMSE) parameter, thus showing that all models produced predictions that were close to the observed data. The results of the *F* test indicate that the predicted and observed values for the models are not significantly different. A non-significant lack of fit *F*-value (*p* > 0.05) was observed, indicating that the variation between samples was due only to the factors selected for the model and the pure error. The *F*-values for model parameters are very useful as indicators of the significance of the effects of the variables and their interactions. In both PEF-treated and the untreated samples, the most significant effect on extraction was the quadratic term of the proportion of hexane (H^2^), followed by time. This means that changes in these factors will have the most significant effect on the carotenoid extraction. The significance of the squared factor (H^2^) reveals the existence of an optimum hexane proportion for the highest CEY at 47% of hexane. Similar results were obtained by Strati (31), who achieved the maximum CEY with a solvent mixture at 45% of hexane.

**Figure 5:**
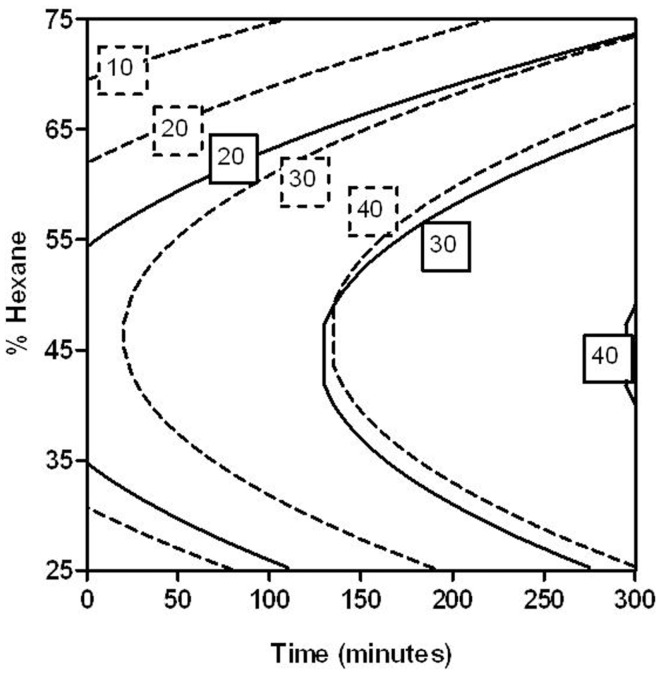
**Combination of percentage of hexane (25–75%) and extraction time (0–300 min) to obtain different carotenoid extraction yields (CEYs) from control (solid lines) and PEF-treated (dotted lines) tomato peels**.

On the other hand, these models enabled the estimation of the conditions (percentage of hexane and extracting time) to achieve a certain CEY from untreated and PEF-treated tomato peels. As an example, Figure 5 shows the combination of extraction time and percentage of hexane for obtaining several CEY from untreated and PEF-treated tomato peels in the investigated range. In this figure, it can be observed that the extraction improvement caused by PEF could allow one to reduce the hexane percentage after a given extraction time to get a fixed CEY. For instance, the percentage of hexane could be reduced from 45 to 30% when the extraction time was set at 150 min by applying a PEF treatment before extraction in order to achieve a CEY of 30 μg/g FW of tomato peel.

More noticeably, from this figure it can be pointed out that the application of PEF treatments would enable one to considerably decrease the extracting time considerably. For instance, the application of a PEF treatment to the tomato peels before extraction could reduce the extracting time more than six times (from 130 to 20 min.) to extract 30 μg/g FW with a 47% of hexane in comparison to the control.

### HPLC analysis of the main carotenoid obtained by extraction from tomato peels treated by PEF

Reverse-phase HPLC chromatogram profiles detected at 472 nm for the extract of untreated and PEF-treated tomato peels were similar for both untreated and treated samples and only depended on the proportion of hexane in the solvent mixture (results not shown). In both untreated and PEF-treated samples, the percentage of individual carotenoids differed depending of the proportion of solvents used in the mixture. The lowest (28%) and the highest percentages (100%) for lycopene were observed in hexane 25% and hexane 75% extracts, respectively. On the other hand, lutein was only observed at proportions of around 18% when the proportion of hexane in the mixture was 25%. These results are consistent with the observation of Strati (31) and Hakala (36). Such findings could be related to the solubility of lutein in ethanol, which is 15-folds higher than the respective one in hexane. Similarly, the solubility of lycopene in ethanol is 20-folds lower than in hexane (37).

**Figure 6:**
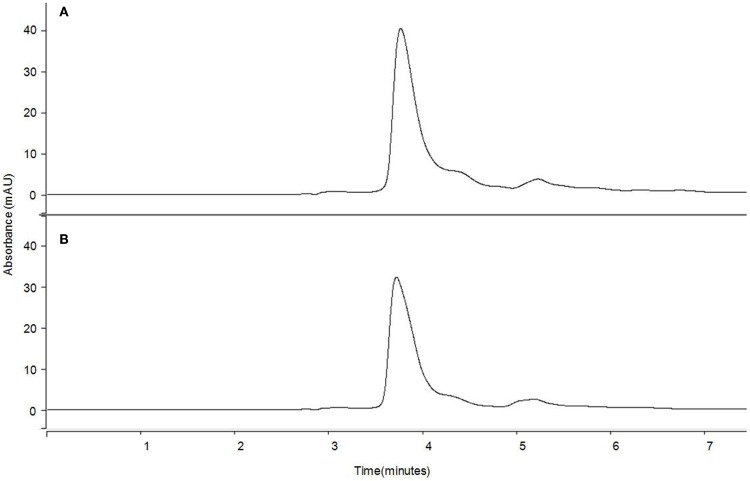
**HPLC chromatograms of carotenoid profiles of 50% hexane extract from tomato peel at 472 nm for (A) PEF-treated and (B) control sample**.

Figure 6 compares as an example the HPLC chromatogram profiles at 472 nm for the extract obtained from untreated and PEF-treated tomato peels in a solvent with the same proportion of hexane and ethanol (50:50). The application of a PEF treatment to the tomato peel before extraction did not specifically affect the extraction of a certain carotenoid and no evidence of carotenoid degradation or isomerization by PEF treatments could be found. The only difference observed was that the peak areas were approximately 20% higher in the chromatograms that corresponded to the PEF-treated tomato peels. Similar results were obtained by other authors, who observed analogous chromatogram profiles of the control and PEF-treated samples, but with bigger peaks in the PEF-treated ones (14, 38, 39).

### Effect of electric field strength on the antioxidant activity of tomato peel extract

**Figure 7:**
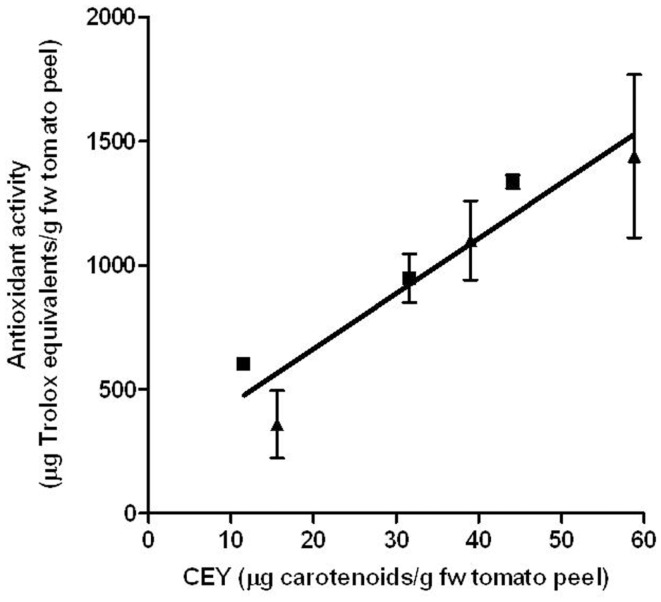
**Relationship between the carotenoid extraction yield (CEY) and antioxidant capacity of the extracts obtained from control (■) and PEF-treated (▲) tomato peels**. The error bars represent SEM.

Beneficial implications of carotenoids in human health are due to their capacity to protect biomolecules against oxidation by quickly reducing reactive oxygen species including free radicals. The effect of PEF treatment on the antioxidant potential of the corresponding extracts was evaluated. As shown in Figure 7, the antioxidant activities of the extracts were related to the amount of extracted carotenoids. No statistically significant differences (*p* > 0.05) were observed in the antioxidant capacity of the carotenoids that were extracted from the control and the one extracted from the PEF-treated tomato peels. Therefore, PEF-assisted extracts had a higher antioxidant capacity than the control ones due to their higher carotenoid concentration. It has been observed that PEF application also increased the antioxidant activity of the grape by-product extracts; further, the apple juice that was extracted by pressing was approximately twofolds higher for the PEF-treated samples than in the control extraction (12, 40). This increment has also been related to the higher concentration of compounds with antioxidant activity in the extracts.

## Concluding Remarks

Generally, the efficacy of PEF for improving the extraction of intracellular compounds has been assessed with water-soluble molecules. The results obtained in this investigation demonstrated that the electroporation of cytoplasmic membranes of tomato peels also increases the extraction of fat-soluble compounds such as carotenoids. The use of a mixture of polar and non-polar solvents results in the highest extraction yield for both untreated and PEF-treated samples. The application of a PEF pre-treatment to the tomato peels before extraction with a mixture of hexane:ethanol permitted an increase in the extraction time or the reduction of the proportion of hexane in the sample and extraction time without affecting the extraction yield.

## Conflict of Interest Statement

The authors declare that the research was conducted in the absence of any commercial or financial relationships that could be construed as a potential conflict of interest.
